# Asthma prevalence and associated factors among lebanese adults: the first national survey

**DOI:** 10.1186/s12890-021-01529-z

**Published:** 2021-05-13

**Authors:** Zeina Akiki, Danielle Saadeh, Rita Farah, Souheil Hallit, Hala Sacre, Hassan Hosseini, Pascale Salameh

**Affiliations:** 1 INSPECT-LB (Institut National de Santé Publique d’Épidémiologie Clinique et de Toxicologie-Liban), Beirut, Lebanon; 2 Faculty of Public Health, Lebanese University, Fanar, Lebanon; 3 School of Pharmacy, Lebanese International University, Beirut, Lebanon; 4 CERIPH, Center for Research in Public Health, Pharmacoepidemiology Surveillance Unit, Faculty of Public Health, Lebanese University, Fanar, Lebanon; 5 Faculty of Pharmacy, Lebanese University, Hadat, Lebanon; 6 Faculty of Medicine and Medical Sciences, Holy Spirit University of Kaslik (USEK), Jounieh, Lebanon; 7 Life Sciences and Health Department, Paris-Est University, Paris, France; 8 Henri Mondor Hospital, Paris, France; 9 University of Nicosia Medical School, Nicosia, Cyprus

**Keywords:** Prevalence, Adults, Physician-diagnosed asthma, Current asthma, Asthma risk factors

## Abstract

**Background:**

No national research has yet explored the prevalence of asthma among adults in Lebanon. This study aims to evaluate the prevalence of physician-diagnosed asthma and current asthma, and their determinants among Lebanese adults 16 years old or above.

**Methods:**

A cross-sectional study was carried out using a multistage cluster sampling. The questionnaire used collected information on asthma, respiratory symptoms, and risk factors.

**Results:**

The prevalence of physician-diagnosed asthma was 6.7% (95% CI 5–8.7%), and that of current asthma was 5% (95% CI 3.6–6.9%). Chronic symptoms such as cough, wheezing, and shortness of breath were worst at night. Factors positively associated with physician-diagnosed asthma were a secondary educational level (adjusted OR, aOR = 4.45), a family history of chronic respiratory diseases (aOR = 2.78), lung problems during childhood (15.9), and allergic rhinitis (4.19). Additionally, consuming fruits and vegetables less than once per week (3.36), a family history of chronic respiratory diseases (3.92), lung problems during childhood (9.43), and allergic rhinitis (8.12) were positively associated with current asthma.

**Conclusions:**

The prevalence of asthma was within the range reported from surrounding countries. However, repeated cross-sectional studies are necessary to evaluate trends in asthma prevalence in the Lebanese population.

**Supplementary Information:**

The online version contains supplementary material available at 10.1186/s12890-021-01529-z.

## Background

Asthma, a devastating global health problem, has been an area of interest and a great challenge for health care professionals worldwide due to its high prevalence, its complexity, heterogeneity, and its substantial burden on individuals, their families, and countries. This chronic inflammatory disease characterized by recurrent episodes of wheezing, cough, shortness of breath, and chest tightness, varying over time and in intensity, affects around 300 million individuals worldwide [1]. Asthma has nearly doubled over the past decades [2] and is expected to hit an additional 100 million people by 2025 [3]. The World Health Survey (WHS) has estimated the prevalence of physician-diagnosed asthma to be 4.3% worldwide, with a variation of up to 21-fold between countries [4]. These differences in asthma prevalence highlight the need for additional studies to allow comparisons and plan interventions. However, while the prevalence of asthma is still increasing in some countries, it has been suggested that the increase in the asthma epidemic is coming to an end in some countries with a high prevalence of the disease [5]. Asthma is associated with different personal and environmental factors, such as the female gender, a history of early lung infections, rhinitis, and obesity [6, 7]. It is estimated that around 200,000 people die prematurely each year as a result of asthma, with over 80% of these deaths occurring in low- and middle-income countries [8, 9].

In Lebanon, previous studies have assessed asthma prevalence in specific age groups, mainly among students 3–15 [10], 5–12 [11], or 13–14 years old [12], and adults 40 years of age or older [13]. Other studies have explored correlated factors [14–18], but to our knowledge, none has assessed asthma prevalence at the national level, and by including adults 16 years old or above. Therefore, conducting an accurate study on the epidemiology of asthma in Lebanon seemed necessary. This national study aimed to evaluate the prevalence of physician-diagnosed asthma, current asthma, and their determinants among Lebanese adults.

## Methods

### Study design and population

A national cross-sectional study was carried out and data collection took place between February and June 2020, using a multistage cluster sampling. First, 70 villages/cities from all the governorates were randomly selected from the list of all the Lebanese villages provided by the Central Agency of Statistics in Lebanon. Then a random sample of 10–11 Lebanese adults 16 years old or above was selected from each village. In total, 770 participants were approached, 36 refused to participate and 734 participants were enrolled (4.7% non-response rate).

### Sample size calculation

The minimum sample size was calculated using the G-Power software version 3.0.10. The calculated effect size was 0.0526, expecting a squared partial multiple correlation coefficient of 0.05 (R2 increase) related to the Omnibus test of multiple regression. The minimum necessary sample was n = 609, considering an alpha error of 5%, a power of 80%, and allowing 50 predictors to be included in the model.

### Data collection

Five well-trained study-independent persons collected data through face-to-face interviews. Arabic, the native language in Lebanon, was used during the interview. The questionnaire consisted of four sections. The first included questions on socio-demographic characteristics, such as age, height, weight, educational level, employment, and place of residence. The second consisted of items describing lifestyle characteristics, such as living conditions (living in a polluted area, working in an industrial area…), eating fruits and vegetables. The third included questions related to the health status, such as respiratory symptoms (cough, expectoration, and wheezing), family history of respiratory diseases, and history of lung problems during childhood. The fourth consisted of items related to smoking status in terms of type (cigarettes or waterpipes), duration, and quantity. The questionnaire required 15 min to complete and was previously used by the researchers who conducted the study [19, 20] (see Additional file 1).

### Respiratory phenotypes

“Physician-diagnosed asthma” was defined by a positive answer to the question: “Has the doctor ever told you that you have a chronic respiratory disease?” with the specification of “asthma” as the type of the aforementioned chronic respiratory disease [4]. “Current asthma” was defined by a report of respiratory symptoms (wheeze, attacks of breathlessness following strenuous activity, at rest or at night time, and asthma attacks) or use of inhaled and/or oral medicines because of breathing problems in the past twelve months to approximate as closely as possible the definition used in the Epidemiological study on the Genetics and Environment of Asthma, bronchial hyper responsiveness and atopy (EGEA) [21]. Allergic rhinitis was assessed by the presence of sneezing, or a runny or blocked nose without having flu [16, 22].

### Cigarette and waterpipe smoking

Current cigarette smokers (CCS) were defined as persons who smoked daily in the past 30 days [23]. In the absence of a standard definition of a regular waterpipe smoker, current waterpipe smokers (CWS) were defined as smoking at least one waterpipe per month [24]. Previous cigarette smokers (PCS) were defined as those who used to be regular cigarette smokers (smoked at least 100 cigarettes in his/her life) but who had quit smoking at the time of the interview [25]. Previous waterpipe smokers (PWS) were defined as those who used to smoke regularly (once per month at least) but have not smoked in more than one month [26, 27].

### Environmental data

Environmental data were self-reported and collected with respect to exposure to indoor and outdoor air pollution and indoor and outdoor allergens. Exposure to indoor air pollution was defined as being exposed to passive or active smoking, or polluting heating devices, such as wood, while outdoor air pollution was described as the exposure to pollutants, such as living in proximity of factories, industries, generators, or biomass exposure. Exposure to indoor allergens was defined as being in the presence of furred pets such as dogs or cats, dust, mites, or carpets, while that to outdoor allergens as being exposed to dust, sand, soil, or the presence of trees on the plot [7, 28].

### Statistical analyses

Descriptive statistics were performed to represent the participants’ characteristics, risk factors, and symptoms and the prevalence of physician-diagnosed asthma and current asthma and were expressed as percentages, means and SD, or prevalence and 95% confidence interval (CI) when applicable.

Moreover, the associations between physician-diagnosed asthma, current asthma, and the participants’ characteristics and risk factors were compared with the chi-square tests and expressed as prevalence and 95% CI. Two multivariable logistic regression models were conducted with physician-diagnosed asthma and current asthma as dependent variables, respectively. The participants’ characteristics or risk factors that had a *P* value of less than 0.2 in the bivariate analysis (such as educational level, smoking, family history of chronic respiratory diseases…) were included as covariates. Adjusted odds ratios (aOR) and their 95% CIs were used to quantify the associations between variables and asthma.

An alpha of 0.05 was used to determine statistical significance. All analyses were performed using IBM’s Statistical Package for the Social Sciences (SPSS) version 22.0 (IBM, Inc, Chicago, IL).

## Results

### Participants’ characteristics

The mean age of the 685 adults without asthma was 40 years, half of them were women, 58% were married, 15% were obese, and 32%, 22%, 5%, and 2% were CCS, CWS, PCS, and PWS respectively.

Participants with physician-diagnosed asthma (n = 49) had a mean age of 36 years, around half of them were women and married, and 13% were obese. Moreover, 60% had a university degree, and 25%, 6%, 10%, and 8% were CCS, CWS, PCS, and PWS, respectively. Additionally, 80% and 63% were exposed to indoor and outdoor air pollution, respectively, and 65% reported having allergic rhinitis.

As for participants with current asthma (n = 37), the mean age was of 37 years, half of them were women, 43% were married, and 11% were obese. Moreover, 56% had a university degree, 63% were currently working, and 16%, 8%, 14%, and 3% were CCS, CWS, PCS, and PWS, respectively. Additionally, 78 and 70% were exposed to indoor and outdoor air pollution, respectively, and 81% reported having allergic rhinitis (Table 1). To note that among asthma patients, 3 reported having a chronic obstructive pulmonary disease (COPD) diagnosed by a physician.

**Table 1:** Participants’ characteristics

	All participants N = 734	Without asthma N = 685	Physician diagnosed asthma N = 49	Current asthma N = 37
*Gender*				
Females %	369 (50.3)	345 (50.4)	24 (49)	19 (51.4)
Age				
n (Mean ± SD)	734 (39.9 ± 15.2)	685 (40.2 ± 15.3)	49 (36 ± 13)	37 (37.0 ± 14.3)
*Age classes*				
[16–35[	309 (42.1)	284 (41.5)	25 (51)	18 (48.6)
[35–55[	286 (39)	266 (38.8)	20 (40.8)	15 (40.5)
55 or above	139 (18.9)	135 (19.7)	4 (8.2)	4 (10.8)
*Body mass index (BMI)*				
< 25 kg/m^2^	308 (42.8)	286 (42.6)	22 (45.8)	19 (51.4)
[25–30[ kg/m^2^	306 (42.5)	286 (42.6)	20 (41.7)	14 (37.8)
≥ 30 kg/m^2^	106 (14.7)	100 (14.9)	6 (12.5)	4 (10.8)
*Residence*				
Beirut	17 (2.4)	16 (2.4)	1 (2.1)	2 (5.6)
Beqaa and Baalbek	128 (17.7)	124 (18.4)	4 (8.3)	4 (10.8)
Mount Lebanon	246 (34.1)	228 (33.8)	18 (37.5)	14 (38.9)
Nabatieh	20 (2.8)	19 (2.8)	1 (2.1)	-
North & Akkar	173 (24)	160 (23.7)	13 (27.1)	14 (38.9)
South	138 (19.1)	127 (18.8)	11 (22.9)	2 (5.6)
*Marital status*				
Single	262 (36.1)	240 (35.5)	22 (44.9)	20 (54.1)
Married	417 (57.4)	392 (57.9)	25 (51)	16 (43.2)
Widowed/Divorced	47 (6.5)	45 (6.6)	2 (4.1)	1 (2.7)
*Education*				
≤ Complementary	148 (20.4)	145 (21.4)	3 (6.3)	5 (13.9)
Secondary	209 (28.8)	193 (28.5)	16 (33.3)	11 (30.6)
University	369 (50.8)	340 (50.1)	29 (60.4)	20 (55.6)
*Occupation*				
Student	69 (9.6)	64 (9.6)	5 (10.6)	3 (8.6)
Currently working	463 (64.6)	430 (64.2)	33 (70.2)	22 (62.9)
Not working	153 (21.3)	144 (21.5)	9 (19.1)	9 (25.7)
Retreat	32 (4.5)	32 (4.8)	-	1 (2.9)
*CCS*				
Yes %	228 (31.1)	216 (31.5)	12 (24.5)	6 (16.2)
*CWS*				
Yes %	151 (20.6)	148 (21.7)	3 (6.1)	3 (8.1)
*PCS*				
Yes %	42 (5.7)	37 (5.4)	5 (10.2)	5 (13.5)
*PWS*				
Yes %	19 (2.6)	15 (2.2)	4 (8.2)	1 (2.7)
*Indoor allergens*				
Yes %	473 (64.4)	440 (64.2)	33 (67.3)	28 (63.6)
*Indoor air pollution*				
Yes %	555 (75.6)	516 (75.3)	39 (79.6)	29 (78.4)
*Outdoor allergens*				
Yes %	104 (14.2)	91 (13.3)	13 (26.5)	10 (27.0)
*Outdoor air pollution*				
Yes %	452 (61.6)	421 (61.5)	31 (63.3)	26 (70.3)
*Fruits and vegetables*				
Once at least day	514 (70.9)	484 (71.6)	30 (61.2)	21 (56.8)
2–3 times/week	141 (19.4)	131 (19.4)	10 (20.4)	8 (21.6)
< than once/week	70 (9.7)	61 (9.0)	9 (18.4)	8 (21.6)
*Family history of chronic respiratory disease*				
Yes %	132 (18.1)	110 (16.1)	22 (44.9)	22 (59.5)
*Lung problems (childhood)*				
Yes %	53 (7.2)	34 (5.0)	19 (38.8)	11 (29.7)
*Allergic rhinitis*				
Yes %	239 (32.6)	207 (30.2)	32 (65.3)	30 (81.1)

All values are expressed as n (%) except for age which is expressed a n (mean ± SD)

*SD* standard deviation, *CCS* Current cigarette smokers, *CWS* current waterpipe smokers, *PCS* previous cigarette smokers, *PWS* previous waterpipe smokers

### Symptoms among participants with current asthma

**Fig. 1:**
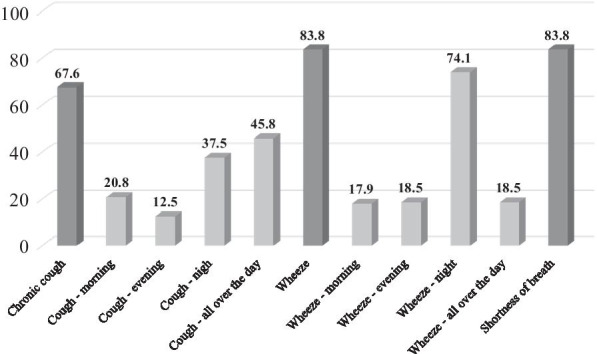
Symptoms among participants with current asthma (in percent): chronic cough, wheezing, and shortness of breath as well as the dayparts during which these symptoms appeared most (morning, evening, night, or all over the day). The horizontal axis shows the symptoms and their time of occurrence during the day. The vertical axis presents the percentages

Among participants with current asthma, 68% reported chronic cough, 84% wheezing, and 84% shortness of breath. As for the dayparts during which these symptoms appeared most, wheezing at night was the most common (74%), followed by cough all over the day (46%), and cough at night (38%) (Fig. 1).

### Prevalence of physician-diagnosed asthma

The prevalence of physician-diagnosed asthma was 6.7% (95% CI 5–8.7%). It was significantly higher among participants with a secondary or university educational level compared to complementary or less, among PWS, participants exposed to outdoor allergens, with a family history of chronic respiratory diseases, with lung problems during childhood, and with allergic rhinitis (*P* values < 0.05). However, it was lower among CWS versus not. No other significant associations were detected. In the multivariable analysis, secondary educational level compared to complementary [aOR (95% CI) = 4.45 (1.14;17.3)], a family history of chronic respiratory diseases [2.78 (1.32;5.83)], lung problems during childhood [15.9 (7.02;35.8)], and allergic rhinitis [4.19 (2.03;8.65)] were positively associated with physician-diagnosed asthma; yet, a negative association was observed with being a CWS [0.17 (0.04;0.67)] (Table 2).

**Table 2:** Prevalence of physician-diagnosed asthma according to the participants’ characteristics and risk factors

	Physician-diagnosed asthma			
	Bivariate analysis		Multivariable analysis-N = 715	
	P (95% CI)	*P* value	aOR (95% CI)	*P* value
	*6.7 (5.0*–*8.7)*			
*Gender*				
Males	6.9 (4.5–10)	0.8	–	
Females	6.5 (4.2–9.5)			
*Age classes*				
[16–35]	8.1 (5.3–11.7)	0.1	Reference	
[35–55]	7.0 (4.3–10.6)		1.01 (0.46; 2.19)	0.9
55 or above	2.9 (0.8–7.2)		0.56 (0.17; 1.87)	0.3
*Body mass index (BMI)*				
< 25 kg/m^2^	7.1 (4.5–10.6)	0.9	–	
[25–30] kg/m^2^	6.5 (4.0–9.9)			
≥ 30 kg/m^2^	5.7 (2.1–11.9)			
*Residence*				
Beirut	5.9 (0.1–28.7)	0.6	–	
Bekaa and Baalback	3.1 (0.9–7.8)			
Mount Lebanon	7.3 (4.4–11.3)			
Nabatieh	5.0 (0.1–24.9)			
North and Akkar	7.5 (4.1–12.5)			
South	8.0 (4.0–13.8)			
*Marital status*				
Single	8.4 (5.3–12.4)	0.4	–	
Married	6.0 (3.9–8.7)			
Widowed/divorced	4.3 (0.5–14.5)			
*Education*				
≤ Complementary	2.0 (0.4–5.8)	**0.04**	Reference	
Secondary	7.7 (4.4–12.1)		4.45 (1.14; 17.3)	**0.03**
University	7.9 (5.3–11.1)		3.83 (0.99; 14.8)	0.05
*Occupation*				
Student	7.2 (2.4–16.1)	0.4	–	
Currently working	7.1 (5.0–9.9)			
Not working	5.9 (2.7–10.9)			
Retired	0			
*CCS*				
No	7.3 (5.2–9.9)	0.3	–	
Yes	5.3 (2.7–9.0)			
*CWS*				
No	7.9 (5.9–10.4)	**0.009**	Reference	**0.01**
Yes	2.0 (0.4–5.7)		0.17 (0.04; 0.67)	
*PCS*				
No	6.4 (4.7–8.4)	0.2	–	
Yes	11.9 (4.0–25.6)			
*PWS*				
No	6.3 (4.6–8.3)	**0.01**	Reference	0.4
Yes	21.1 (6.1–45.6)		1.95 (0.46; 8.35)	
*Indoor allergens*				
No	6.1 (3.5–9.8)	0.7	–	
Yes	7.0 (4.9–9.7)			
*Indoor air pollution*				
No	5.6 (2.7–10)	0.5	–	
Yes	7.0 (5.0–9.5)			
*Outdoor allergens*				
No	5.7 (4.0–7.8)	**0.01**	Reference	0.2
Yes	12.5 (6.8–20.4)		1.77 (0.75; 4.17)	
*Outdoor air pollution*				
No	6.4 (3.8–9.9)	0.8	–	
Yes	6.9 (4.7–9.6)			
*Fruits and vegetables*				
Once at least daily	5.8 (4.0–8.2)	0.09	Reference	
2–3 times/week	7.1 (3.5–12.7)		0.89 (0.36; 2.24)	0.8
< than once/week	12.9 (6.1–23.0)		2.28 (0.90; 5.77)	0.08
*Family history of chronic respiratory disease*				
No	4.5 (3.0–6.5)	** < 0.001**	Reference	**0.007**
Yes	16.7 (10.7–24.1)		2.78 (1.32; 5.83)	
*Lung problems during childhood*				
No	4.4 (3.0–6.2)	** < 0.001**	Reference	** < 0.001**
Yes	35.8 (23.1–50.2)		15.9 (7.02; 35.8)	
*Allergic rhinitis*				
No	3.4 (2.0–5.4)	** < 0.001**	Reference	** < 0.001**
Yes	13.4 (9.3–18.4)		4.19 (2.03; 8.65)	

Bold numbers represent significant results (*p* < 0.05)

R^2^ = 35.0; Hosmer & Lemshow–*P* value = 0.468; 93.6% of the participants correctly classified

*P* prevalence, *aOR* adjusted Odds ratio, *CI* confidence interval, *CCS* current cigarette smokers, *CWS* current waterpipe smokers, *PCS* previous cigarette smokers, *PWS* previous waterpipe smokers

### Prevalence of current asthma

The prevalence of current asthma was 5% (95% CI 3.6–6.9%). It was significantly higher among PCS, participants exposed to outdoor allergens, those consuming fruits and vegetables less than once per week, participants with a family history of chronic respiratory diseases, with lung problems during childhood, and with allergic rhinitis (*P* values < 0.05). However, the prevalence was lower among CCS, CWS, and persons living in the South compared to Beirut. No other significant associations were observed. In the multivariable analysis, the consumption of fruits and vegetables less than once per week [aOR (95% CI) = 3.36 (1.14–9.92)], a family history of chronic respiratory diseases [3.92 (1.73–8.92)], lung problems during childhood [9.43 (3.47–25.6)], and allergic rhinitis [8.12 (3.07–21.5)] were positively associated with current asthma while living in the South [0.05 (0.004–0.51)] and being a CCS showed a negative association [0.31 (0.11–0.87)] (Table 3).

**Table 3:** Prevalence of current asthma according to the participants’ characteristics and risk factors

	Current asthma			
	Bivariate analysis		Multivariable analysis-N = 711	
	P (95% CI)	*P* value	aOR (95% CI)	*P* value
	*5.0 (3.6*–*6.9)*			
*Gender*				
Males	4.9 (3.0–7.7)	0.9	–	
Females	5.1 (3.1–7.9)			
*Age classes*				
[16–35]	5.8 (3.5–9.1)	0.4	–	
[35–55]	5.2 (3.0–8.5)			
55 or above	2.9 (0.8–7.2)			
*Body ma3ss index (BMI)*				
< 25 kg/m^2^	6.2 (3.8–9.5)	0.5	–	
[25–30] kg/m^2^	4.6 (2.5–7.6)			
≥ 30 kg/m^2^	3.8 (1.0–9.4)			
*Residence*				
Beirut	11.8 (1.5–36.4)	**0.05**	Reference	
Beqaa and Baalbek	3.1 (0.9–7.8)		0.12 (0.01–1.10)	0.06
Mount Lebanon	5.7 (3.1–9.4)		0.20 (0.03–1.36)	0.1
Nabatieh	0		–	
North and Akkar	8.1 (4.5–13.2)		0.18 (0.03–1.29)	0.09
South	1.4 (0.2–5.1)		0.05 (0.004–0.51)	**0.01**
Marital status				
Single	6.1 (3.5–9.7)	0.5	–	
Married	4.8 (3.0–7.3)			
Widowed/divorced	2.1 (0.1–11.3)			
*Education*				
≤ Complementary	3.4 (1.1–7.7)	0.6	–	
Secondary	5.3 (2.7–9.2)			
University	5.4 (3.3–8.2)			
*Occupation*				
Student	4.3 (0.9–12.2)	0.9	–	
Currently working	4.8 (3.0–7.1)			
Not working	5.9 (2.7–10.9)			
Retired	3.1 (0.1–16.2)			
*CCS*				
No	6.1 (4.2–8.6)	**0.05**	Reference	**0.03**
Yes	2.6 (1.0–5.6)		0.31 (0.11–0.87)	
*CWS*				
No	5.9 (4.1–8.1)	**0.05**	Reference	0.06
Yes	2.0 (0.4–5.7)		0.25 (0.06–1.05)	
*PCS*				
No	4.6 (3.2–6.5)	**0.05**	Reference	0.7
Yes	11.9 (4.0–25.6)		1.29 (0.34–4.93)	
*PWS*				
No	5.0 (3.6–6.9)	0.9	–	
Yes	5.3 (0.1–26.0)			
*Indoor allergens*				
No	5.0 (2.7–8.4)	0.9	–	
Yes	5.1 (3.3–7.5)			
*Indoor air pollution*				
No	4.5 (1.9–8.6)	0.7		
Yes	5.2 (3.5–7.4)			
Outdoor allergens				
No	4.3 (2.8–6.2)	**0.02**	Reference	0.3
Yes	9.6 (4.7–17.0)		1.72 (0.64–4.61)	
*Outdoor air pollution*				
No	3.9 (2.0–6.9)	0.3	–	
Yes	5.8 (3.8–8.3)			
*Fruits and vegetables*				
Once at least daily	4.1 (2.5–6.2)	**0.03**	Reference	
2–3 times/week	5.7 (2.5–10.9)		1.31 (0.48–3.57)	0.6
< than once/week	11.4 (5.1–21.3)		3.36 (1.14–9.92)	**0.03**
*Family history of chronic respiratory disease*				
No	2.5 (1.4–4.1)	** < 0.001**	Reference	**0.001**
Yes	16.7 (10.7–24.1)		3.92 (1.73–8.92)	
*Lung problems during childhood*				
No	3.8 (2.5–5.5)	** < 0.001**	Reference	** < 0.001**
Yes	20.8 (10.8–34.1)		9.43 (3.47–25.6)	
*Allergic rhinitis*				
No	1.4 (0.6–2.9)	** < 0.001**	Reference	** < 0.001**
Yes	12.6 (8.6–17.4)		8.12 (3.07–21.5)	

Bold numbers represent significant results (*p* < 0.05)

R^2^ = 38.9; Hosmer & Lemshow–* P* value = 0.912; 95.1% of the participants correctly classified

*P* prevalence, *aOR* adjusted Odds ratio, *CI* confidence interval, *CCS* current cigarette smokers, *CWS* current waterpipe smokers, *PCS* previous cigarette smokers, *PWS* previous waterpipe smokers

## Discussion

To our knowledge, this study is the first national study to evaluate asthma prevalence among Lebanese adults 16 years old or above. The prevalence of physician-diagnosed asthma was 6.7%, and that of current asthma 5%. Participants with current asthma reported symptoms of chronic cough, wheezing, or shortness of breath that mainly appeared at night. A secondary educational level, a family history of chronic respiratory diseases, lung problems during childhood, and allergic rhinitis, were significantly and positively associated with physician-diagnosed asthma. Moreover, the consumption of fruits and vegetables less than once per week, a family history of chronic respiratory diseases, lung problems during childhood, and allergic rhinitis, were positively associated with current asthma.

The present study revealed a prevalence of physician-diagnosed asthma of 6.7%. Studies assessing the prevalence of physician-diagnosed asthma show that it varies in different parts of the world. The WHS conducted among 178,215 adults from 70 countries estimated the prevalence of physician-diagnosed asthma at 4.3% worldwide, ranging from 0.2% (in China) to 21.0% (in Australia) [4]. Another study conducted in Iran in 2018 among participants 20–44 years old revealed a prevalence of physician-diagnosed asthma of 3.7% [29]. It seems that the prevalence of physician-diagnosed asthma in Lebanon is higher than the overall prevalence reported by the WHS or that reported in Iran. However, the current literature is unclear about the exact prevalence of asthma, showing a substantial variation worldwide. Presumably, this is due to variations in diagnostic criteria, the inclusion of specific age groups, and gene-environmental interactions [28]. Nevertheless, our prevalence is lower than that reported in Saudi Arabia of 11.3% among adults between 20 and 40 years old [30]. In the latter study, the authors used the European Community Respiratory Health Survey (ECRHS) questionnaire in the adult Saudi population without validation, which might have led to measurement errors, as reported by Al Ahmari [31]. The prevalence of physician-diagnosed asthma in our study is higher than that reported among students 5–12 years old (4.8%) [11] and lower than the prevalence of ever asthma among students 13–14 years old (8.3%) [12]. Although it is not reliable to compare the prevalence of asthma in adults to that found among children, we believe that recently there is an increase in physicians’ awareness of asthma disease in Lebanon. Additionally, a previous study had reported a prevalence of physician-diagnosed asthma among Lebanese adults 40 years old or above of 4.6% in 2014 [13]. Lebanon has suffered from rising pollution, especially after the waste crisis in 2015, which might explain this increase in the prevalence of asthma among adults.

The present study revealed that the prevalence of current asthma was 5%. In 2006, the prevalence of current asthma in France, defined by the presence of asthma symptoms or the use of asthma medication in the past 12 months, was 7% [32]. In Iran, the pooled prevalence of current asthma, defined as a history of one or more attack of dyspnea and wheezing during the past 12 months, was 8.8% [33], and the prevalence of current asthma, defined as the presence of either an attack of shortness of breath, an attack of asthma, or use of asthma medication, was 4.7% [29]. Additionally, a study conducted in Turkey in 2014 reported a prevalence of current asthma, defined as having an asthma attack and/or treatment for asthma in the past 12 months, of 6.9% [34]. Although our prevalence is within the range reported from surrounding countries, possible interpretations of the variation include the changing in the definition of current asthma across many study settings and the effect of gene-environment interactions [35]. A study conducted among Lebanese adults 40 years or above reported a prevalence of hyperreactive airways at 9.9% [13]. In our study, the prevalence of current asthma was estimated according to the most up-to-date definition; therefore, we believe that it reflects the real situation among Lebanese adults. Moreover, our results showed a prevalence of current asthma lower than that of physician-diagnosed asthma, although the former is more sensitive while the latter is more specific. Sensitive definitions might yield a greater asthma prevalence [35]. In this regard, it is important to note that this increase in prevalence could be due to increased physician awareness of asthma and better diagnostic practices and management strategies.

The factors that were commonly associated with physician-diagnosed asthma and current asthma were a family history of chronic respiratory diseases, lung problems during childhood, and allergic rhinitis. The positive association between allergic rhinitis and asthma was repeatedly found in the literature [22, 36, 37]. Moreover, the positive associations between a family history of chronic respiratory diseases and lung problems during childhood and asthma were expected and are in line with previous studies conducted to determine the prevalence and determinants of asthma [13, 36]. Thus, special attention should be given to health problems during childhood, especially respiratory ones.

Our results showed that a moderate educational level (secondary) was positively associated only with physician-diagnosed asthma, in contrast with those of a previous study reporting a higher prevalence of asthma among participants with a low educational level [38]. This discrepancy might be due to the differences in educational levels among both populations. In our study, asthma patients had a higher level of education, thought to be associated with more awareness of asthma symptoms, which could lead to seeking medical advice.

Moreover, consuming fruits less than once per week was positively associated only with current asthma, consistent with the literature showing that the consumption of fruits or vegetables was inversely associated with current asthma and the risk of asthma in adults [39, 40]. Indeed, oxidative stress plays an essential role in the pathophysiology of asthma due to chronic activation of airway inflammatory cells [41]. Since fresh fruits and vegetables are rich sources of antioxidants [42], they can reduce airway inflammation by protecting the airways against both endogenous (activated inflammatory cells) and exogenous (such as air pollution, cigarette smoke) oxidants [41]. Thus, there is a need to promote a healthy diet, especially among asthma patients.

Our study found negative associations between CWS and physician-diagnosed asthma, and between CCS and current asthma, despite the evidence that asthma prevalence is higher among smokers [35]. It is worth to mention that the prevalence of CWS in our sample was very low (6% among physician-diagnosed asthma), which might have affected our findings. Moreover, participants with current asthma were light smokers, not heavy ones. Also, consistent with previous reports conducted in Poland and in Iran [38, 43], living in the South, which is a rural area, was a protective factor for having current asthma.

Furthermore, the present study could not find an association between gender and asthma, which is in line with previous studies [38, 44]. In contrast, a review conducted in 2011 reported that women were more likely to be diagnosed with asthma due to their lower quality of life [45], which we believe is not the case in the Lebanese population. Moreover, research has shown that even though asthma is more prevalent among women, different factors confound the association between gender and asthma, such as aging, obesity, and gender differences in behavior and exposures; thus, additional studies are necessary to confirm the effect of gender on asthma [46]. Although not shown to be independently associated with asthma in the multivariable models, outdoor allergens were significantly associated with asthma in the bivariate analyses. To note that, based on a review published in 2019, exposure to high concentrations of outdoor allergens is mainly linked to asthma exacerbation and mortality in adults, not with the presence of asthma [35].

## Limitations and strengths

Our study is cross-sectional; thus, any temporal relationship between the factors examined and the outcome cannot be established. However, since our main objective was to evaluate the prevalence of asthma, the use of a cross-sectional design seemed necessary. Moreover, information was self-reported, which might include the possibility of information bias. Additionally, we were not able to confirm the diagnosis of asthma with lung function testing because of participants’ anonymity in the study design. However, we believe that the diagnosis was reasonably accurate, as we relied on two elements, the physician diagnosis and the most up-to-date definition for current asthma. In addition, it was not possible to compare respondents to non-respondents due to the lack of information on non-respondents. However, the non-response rate was low (4.7%) and the general characteristics of our sample were similar to those of the Lebanese population as reported by the Central of Administration of Statistics (CAS) (http://www.cas.gov.lb/).

To our knowledge, this is the first national study conducted among a representative sample drawn from all Lebanese districts and exploring asthma prevalence among Lebanese adults 16 years old or above. Our results can be generalized to the Lebanese adult population since our sample is random.

## Conclusions

Asthma prevalence among Lebanese adults was within the range reported from surrounding countries. With increasing urbanization, an aging population, the adoption of Western lifestyles, and the economic crisis, the prevalence of asthma tends to increase in the future. Repeated cross-sectional studies are necessary to evaluate trends in asthma prevalence and assess the asthma burden in Lebanon.

## Declarations

## Supplementary Information

**Additional file 1**. Questionnaire of the study. The questionnaire, in the English language version, used in this present study to collect information on asthma, respiratory symptoms, and risk factors.

## Data Availability

All relevant data for this study are included in this published article and its supplementary information file.

## Abbreviations

WHS: World Health Survey
EGEA: epidemiological study on the genetics and environment of asthma, bronchial hyper responsiveness and atopy
CCS: current cigarette smokers
CWS: current waterpipe smokers
PCS: previous cigarette smokers
PWS: previous waterpipe smokers
CI: confidence interval
aOR: adjusted odds ratios
SPSS: statistical package for the social sciences
COPD: chronic obstructive pulmonary disease
SD: standard deviation
BMI: body mass index
P: prevalence
ECRHS: European Community Respiratory Health Survey
INSPECT-LB: Institut National de Santé Publique, Epidémiologie Clinique et Toxicologie-Liban, Beirut, Lebanon

## References

[1] Bousquet J, Mantzouranis E, Cruz AA, Aït-Khaled N, Baena-Cagnani CE, Bleecker ER (2010). Uniform definition of asthma severity, control, and exacerbations: document presented for the World Health Organization Consultation on Severe Asthma. J Allergy Clin Immunol.

[2] Antó JM (2012). Recent advances in the epidemiologic investigation of risk factors for asthma: a review of the 2011 literature. Curr Allergy Asthma Rep.

[3] Schluger NW, Koppaka R (2014). Lung disease in a global context. A call for public health action. Ann Am Thorac Soc..

[4] To T, Stanojevic S, Moores G, Gershon AS, Bateman ED, Cruz AA (2012). Global asthma prevalence in adults: findings from the cross-sectional world health survey. BMC Public Health.

[5] Eder W, Ege MJ, von Mutius E (2006). The asthma epidemic. N Engl J Med.

[6] Wu TD, Brigham EP, McCormack MC (2019). Asthma in the primary care setting. Med Clin N Am.

[7] Obel KB, Ntumba KJM, Kalambayi KP, Zalagile AP, Kinkodi KD, Munogolo KZ (2017). Prevalence and determinants of asthma in adults in Kinshasa. PloS ONE.

[8] Anandan C, Nurmatov U, van Schayck OCP, Sheikh A (2010). Is the prevalence of asthma declining?. Syst Rev Epidemiol Stud Allergy.

[9] Pawankar R, Canonica GW, Holgate ST, Lockey RF (2012). Allergic diseases and asthma: a major global health concern. Curr Opin Allergy Clin Immunol.

[10] Hallit S, Salameh P (2017). Exposure to toxics during pregnancy and childhood and asthma in children: A pilot study. J Epidemiol Glob Health.

[11] Waked M, Salameh P (2008). Asthma, allergic rhinitis and eczema in 5–12-year-old school children across Lebanon. Public Health.

[12] Musharrafieh U, Al-Sahab B, Zaitoun F, El-Hajj MA, Ramadan F, Tamim H (2009). Prevalence of asthma, allergic rhinitis and eczema among Lebanese adolescents. J Asthma Off J Assoc Care Asthma.

[13] Salamé J, Tyan P, Salameh P, Waked M (2014). Hyperreactive airway disease in adults: data from a national study in Lebanon. J Med Liban.

[14] Waked M, Salameh P (2008). Risk factors for asthma and allergic diseases in school children across Lebanon. J Asthma Allergy.

[15] Salameh P, Karaki C, Awada S, Rachidi S, Al Hajje A, Bawab W (2015). Asthma, indoor and outdoor air pollution: a pilot study in Lebanese school teenagers. Rev Mal Respir.

[16] Waked M, Salameh P (2006). Asthma, allergic rhinitis and eczema in 13–14-year-old schoolchildren across Lebanon. J Med Liban.

[17] Khadadah M, Mahboub B, Al-Busaidi NH, Sliman N, Soriano JB, Bahous J (2009). Asthma insights and reality in the Gulf and the near East. Int J Tuberc Lung Dis Off J Int Union Tuberc Lung Dis.

[18] Bahous J, Soriano JB (2010). Asthma control in Lebanon the asthma insights and reality in Lebanon. J Med Liban.

[19] Akiki Z, Fakih D, Jounblat R, Chamat S, Waked M, Holmskov U (2016). Surfactant protein D, a clinical biomarker for chronic obstructive pulmonary disease with excellent discriminant values. Exp Ther Med.

[20] Fakih D, Akiki Z, Junker K, Medlej-Hashim M, Waked M, Salameh P (2018). Surfactant protein D multimerization and gene polymorphism in COPD and asthma. Respirology.

[21] Kauffmann F, Dizier MH, Pin I, Paty E, Gormand F, Vervloet D (1997). Epidemiological study of the genetics and environment of asthma, bronchial hyperresponsiveness, and atopy: phenotype issues. Am J Respir Crit Care Med.

[22] Bousquet J, Khaltaev N, Cruz AA, Denburg J, Fokkens WJ, Togias A (2008). Allergic Rhinitis and its Impact on Asthma (ARIA) 2008 update (in collaboration with the World Health Organization, GA(2)LEN and AllerGen). Allergy.

[23] Pärna K, Pürjer M-L, Ringmets I, Tekkel M (2014). Educational differences in cigarette smoking among adult population in Estonia, 1990–2010: Does the trend fit the model of tobacco epidemic?. BMC Public Health.

[24] Hallit S, Hallit R, Haddad C, Youssef L, Zoghbi M, Costantine R (2019). Previous, current, and cumulative dose effect of waterpipe smoking on LDL and total cholesterol. Environ Sci Pollut Res Int.

[25] NHIS - Adult Tobacco Use - Glossary [Internet]. 2019 [cited 2020 Sep 4]. Available from: https://www.cdc.gov/nchs/nhis/tobacco/tobacco_glossary.htm

[26] Akiki Z, Saadeh D, Haddad C, Sacre H, Hallit S, Salameh P (2020). Knowledge and attitudes toward cigarette and narghile smoking among previous smokers in Lebanon. Environ Sci Pollut Res Int.

[27] Borgan SM, Jassim G, Marhoon ZA, Almuqamam MA, Ebrahim MA, Soliman PA (2014). Prevalence of tobacco smoking among health-care physicians in Bahrain. BMC Public Health.

[28] 2020 GINA Main Report [Internet]. Global Initiative for Asthma - GINA. [cited 2020 Jul 4]. Available from: https://ginasthma.org/gina-reports/

[29] Fazlollahi MR, Najmi M, Fallahnezhad M, Sabetkish N, Kazemnejad A, Bidad K (2018). The prevalence of asthma in Iranian adults: The first national survey and the most recent updates. Clin Respir J.

[30] Al Ghobain M, Algazlan S, Oreibi T (2018). Asthma prevalence among adults in Saudi Arabia. Saudi Med J.

[31] AlAhmari M (2018). Asthma prevalence among adults in Saudi Arabia. Saudi Med J.

[32] Afrite A, Allonier C, Com-Ruelle L, Guen NL, Annesi-Maesano I, Delmas M-C, et al. L’asthme en France en 2006: prévalence et contrôle des symptômes. 2006;8.

[33] Varmaghani M, Farzadfar F, Sharifi F, Rashidian A, Moin M, Moradi-Lakeh M (2016). Prevalence of Asthma, COPD, and chronic bronchitis in Iran: a systematic review and meta-analysis. Iran J Allergy Asthma Immunol.

[34] Uğurlu E, Öncel SB, Evyapan F (2014). Symptom prevalence and risk factors for asthma at the rural regions of Denizli. Turkey J Thorac Dis.

[35] Dharmage SC, Perret JL, Custovic A. Epidemiology of Asthma in Children and Adults. Front Pediatr [Internet]. 2019 Jun 18 [cited 2020 Sep 16];7. Available from: https://www.ncbi.nlm.nih.gov/pmc/articles/PMC6591438/10.3389/fped.2019.00246 PMC6591438 31275909

[36] Ozoh OB, Aderibigbe SA, Ayuk AC, Desalu OO, Oridota OE, Olufemi O (2019). The prevalence of asthma and allergic rhinitis in Nigeria: a nationwide survey among children, adolescents and adults. PLoS ONE..

[37] Khan DA (2014). Allergic rhinitis and asthma: epidemiology and common pathophysiology. Allergy Asthma Proc.

[38] Idani E, Raji H, Madadizadeh F, Cheraghian B, Haddadzadeh Shoshtari M, Dastoorpoor M (2019). Prevalence of asthma and other allergic conditions in adults in Khuzestan, southwest Iran, 2018. BMC Public Health.

[39] Uddenfeldt M, Janson C, Lampa E, Leander M, Norbäck D, Larsson L (2010). High BMI is related to higher incidence of asthma, while a fish and fruit diet is related to a lower- Results from a long-term follow-up study of three age groups in Sweden. Respir Med.

[40] Woods RK, Walters EH, Raven JM, Wolfe R, Ireland PD, Thien FCK (2003). Food and nutrient intakes and asthma risk in young adults. Am J Clin Nutr.

[41] Tabak C, Wijga AH, de Meer G, Janssen NAH, Brunekreef B, Smit HA (2006). Diet and asthma in Dutch school children (ISAAC-2). Thorax.

[42] Greene LS (1999). Asthma, oxidant stress, and diet. Nutr Burbank Los Angel Cty Calif.

[43] Epidemiology of asthma in Poland in urban and rural areas, based on provided health care services. | Semantic Scholar [Internet]. [cited 2020 Sep 17]. Available from: https://www.semanticscholar.org/paper/Epidemiology-of-asthma-in-Poland-in-urban-and-rural-%C5%9Aliwczy%C5%84ski-Brzozowska/eef43ea01924755e8a22446b30eb0d5a5f2101cd 10.5603/PiAP.2015.0029 26050977

[44] Backman H, Räisänen P, Hedman L, Stridsman C, Andersson M, Lindberg A (2017). Increased prevalence of allergic asthma from 1996 to 2006 and further to 2016—results from three population surveys. Clin Exp Allergy.

[45] Kynyk JA, Mastronarde JG, McCallister JW (2011). Asthma, the sex difference. Curr Opin Pulm Med.

[46] Zein JG, Erzurum SC (2015). Asthma is different in women. Curr Allergy Asthma Rep.

