# Supplementary material for: Asthma prevalence and associated factors among lebanese adults: the first national survey
Source: BMC Pulm Med. 2021 May 13;21:162. doi: 10.1186/s12890-021-01529-z (PMC8120928; doi:10.1186/s12890-021-01529-z)
Supplement: Supplementary file 1 — Additional file 1. Questionnaire of the study. The questionnaire, in the English language version, used in this present study to collect information on asthma, respiratory symptoms, and risk factors. [file 12890_2021_1529_MOESM1_ESM.pdf]

Number: ______________________________

☐ **COPD. Severity:** ☐ **Chronic bronchitis**

☐ **Asthma. Severity:** ☐ **Control**

Part I: Sociodemographic and General Information

Age: _________ years

Gender: ☐ Male ☐ Female

Weight: _________ Kg

Height: _________ cm

Are you: ☐ Married ☐ Single ☐ Widowed or divorced

Education	Work
☐ Never been to school	☐ Currently working
☐ Primary or less	☐ Retired
☐ Complementary or less	☐ Looking for a job
☐ Secondary or less	☐ Never worked
☐ University	☐ Student

Current employment with duration and exposure toxic fumes, gases, or pesticides:

Current Employment	Duration in years	Exposure	
		☐ No	☐ Yes
		☐ No	☐ Yes
		☐ No	☐ Yes
		☐ No	☐ Yes

Number of rooms at home, excluding kitchen and bathroom(s): ___________

Number of persons living with you at home? ___________

Do you have a pet at home? ☐ No ☐ Yes; If yes, specify _________________

Where do you live? __________________

Is it: ☐ A big city ☐ A village ☐ Intermediate

Is your house near a busy road with cars (≤100 meters)? ☐ Yes ☐ No

Have you ever lived in a house near a busy road with cars (≤100 meters)? ☐ Yes ☐ No

If yes, specify for how long: ________________

Have you ever lived in a house near a generator (≤100 meters)? ☐ Yes ☐ No

If yes, specify for how long: ________________

How do you heat your house? ☐ Gas ☐ Wood ☐ Diesel ☐ Electricity ☐ Hot air ☐ Central heating

What do you use to cook? ☐ Gas; If other, specify: _______________

Do you usually burn incense at home? ☐ Yes ☐ No

How many smokers living in your house? ____ **Do they smoke indoors?** ☐ Yes ☐ No

What is the number of smokers at your workplace? ________

Do you get exposed to their cigarette smoke? ☐ No ☐ Yes; For how long (in hours)? _______

Do you eat fruits and vegetables: ☐ at all meals ☐ once per day at least ☐ 2-3 times per week ☐ once per week or less ☐ I do not eat fruits and vegetables at all.

What was your birth weight (grams)? ☐ < 1500 ☐ 1500-1999 ☐ 2000-2499 ☐ 2500-3499 ☐ > 3500 ☐ I don't know.

Were you born within 3 weeks of your due date?

☐ Yes ☐ No, more than 3 weeks before the due date ☐ I don't know

Were you breastfed? ☐ Yes ☐ No

Did you attend kindergarten? ☐ No ☐ Yes; If yes, from what age? ____ Years

Do you have a housemaid? ☐ Yes ☐ No

Do you have a vacuum cleaner at home? ☐ Yes ☐ No

Is there a carpet/rug in your bedroom? ☐ Yes ☐ No

Do you have your own bed? ☐ Yes ☐ No

Is there any apparent humidity on your bedroom walls? ☐ Yes ☐ No

On what kind of pillow do you sleep? ☐ Sponge ☐ Feather ☐ Cotton ☐ Other:__________

On what kind of mattress do you sleep? ☐ Wool ☐ Cotton ☐ Synthetic

Do you think you are exposed to pollution or live in a polluted area?

☐ a lot ☐ a little ☐ never

Do you get exposed to black smoke from cars and motors? ☐ a lot ☐ a little ☐ never

Is the area where you live or work crowded with cars and trucks? ☐ a lot ☐ a little ☐ never

Is the area where you live or work covered in smog in the summer? ☐ a lot ☐ a little ☐ never

Do you get exposed to sand, soil, and dust at work or in any other place?

☐ a lot ☐ a little ☐ never

Do you live or work in an industrial area? ☐ No ☐ Yes; If yes, specify the type of industry and the distance that separates it from your home or workplace: ________________________

Do you live or work next to a power plant? ☐ No ☐ Yes; If yes, specify what power plant and the distance that separates it from your home or workplace: ___________________________

Do you drive a car? ☐ Yes ☐ No

How many hours a day do you spend in transportation (your car or public transport)? _____

Is this transportation conditioned? ☐ Yes ☐ No

Do you have any chronic disease? ☐ No ☐ Yes; If yes, specify: ________________

What medications do you take to treat this diseases? ___________________________________

How do you describe your health from 0 (very bad) to 10 (excellent)? ______________

Does anyone in your family (parents, siblings, or children) have any chronic respiratory disease? ☐ No ☐ Yes; If yes, specify: _________________

Does anyone in your family (parents, siblings, or children) have any type of cancer? ☐ No ☐ Yes; If yes, specify: _________________

Did your mother smoke during pregnancy? ☐ Yes ☐ No ☐ I don't know

Did your father smoke during your mother's pregnancy? ☐ Yes ☐ No ☐ I don't know

Did your mother smoke during your childhood? ☐ Yes ☐ No ☐ I don't know

Did your father smoke during your childhood? ☐ Yes ☐ No ☐ I don't know

Part II: Information on Respiratory Diseases

Has the doctor ever told you that you have a chronic respiratory disease?
☐ No ☐ Yes; If yes, specify________________ (asthma, chronic bronchitis, allergy, COPD)

How old were you when you were diagnosed with this chronic respiratory disease? _______

Are you still suffering from this chronic respiratory disease? ☐ Yes ☐ No

What medications are you taking for this respiratory disease?
____
____

Do you have any chronic respiratory symptoms? ☐ Yes ☐ No

Do you have any chronic cough? ☐ No ☐ Yes; Number of coughing days per week? ______

Since when? ______ **When does it occur?** ☐ in the morning ☐ in the evening ☐ at night ☐ all day

Do you have any chronic expectorations? ☐ No ☐ Yes; Number of expectoration days per week? ______ **Since when?** ______

When does it occur? ☐ in the morning ☐ in the evening ☐ at night ☐ all day

What is the color of sputum? ☐ white ☐ yellow ☐ green ☐ brown

Do you suffer from morning cough with sputum for more than 3 months per year for 2 years or more? ☐ Yes ☐ No

Was your chest congested with sputum or were you spitting sputum for more than 4 days a week and for more than 3 months a year? ☐ Yes ☐ No

Duration in years of these periods? ____ year(s) ☐ never happened

Are there periods in the year (more than 3 weeks) when you have more cough and sputum than usual? ☐ No ☐ Yes; If yes, when do these periods occur? _____________

For how many years did these periods occur? ________

Do you have any wheezing? ☐ Yes ☐ No.

Does this wheezing occur only with flu (cold)? ☐ Yes ☐ No, always

How many times per week do you have wheezing? ___________

Since when? ______ **When?** ☐ in the morning ☐ in the evening ☐ at night ☐ all day

How many times have you wheezed in the past 12 months?

☐ none ☐ 1-3 times ☐ 4-12 times ☐ more than 12 times

In the last 12 months, did you had wheezing during or after sports? ☐ Yes ☐ No

In the last 12 months, what aggravated or trigger wheezing?

☐ weather ☐ pollen ☐ nervousness ☐ smoke ☐ dust ☐ animals ☐ wool ☐ flu ☐ sport

☐ cigarette smoke ☐ foods/drinks ☐soap/medications ☐pollution ☐ other: _______________

☐ I did not wheeze

In the past 12 months, have you had a dry cough at night, without flu or bronchitis?

☐ Yes ☐ No

In the past 12 months, have you had sputum or spitted sputum without flu? ☐ Yes ☐ No

In the past 3 years, did you have to miss work because of a respiratory problem?

☐ Yes ☐ No ☐ I don't know

When you have the flu, does it turn into a lung problem? ☐ No ☐ Yes, most of the times

Do you have any chronic allergy? ☐ No ☐ Yes; If yes, specify to what: _______________

What are the symptoms of this allergy? ☐ dermatologic ☐ nasal ☐ respiratory (wheezing)

Is there any specific time of year when this allergy occurs? ☐ No ☐ Yes; If yes, when _______

Have you ever had sneezing, or bleeding, or nose congestion, without flu? ☐ Yes ☐ No

In the past 12 months, did your nose problem occur with tears or eye problems? ☐ Yes ☐ No

In which of the following months you had your nose problem? ◻ **never happened**

◻ January ◻ February ◻ March ◻ April ◻ May ◻ June

◻ July ◻ August ◻ September ◻ October ◻ November ◻ December

In the past 12 months, did you use any medications, such as pills or spray for wheezing or asthma? ☐ Yes ☐ No

Have you ever had any of the following problems?

Measles ☐ No ☐ Yes; Age ____________

Whooping cough ☐ No ☐ Yes; Age ____________

Have you ever had repetitive otitis? ☐ Yes ☐ No

Have you ever had amygdalectomy? ☐ Yes ☐ No

Do you have any cardiac problem? ☐ Yes ☐ No

When you were born, did you stay in the hospital longer than usual? ☐ Yes ☐ No

Have you recently lost weight (more than 10% in 6 months or 5% in a month) for no reason? ☐ Yes ☐ No

Was your birth weight less than normal? ☐ Yes ☐ No ☐ I don't know

Have you had a chronic lung problem during childhood? ☐ No ☐ I don't know ☐ Yes, specify: ____________________

Have you had pneumonia during childhood? ☐ No ☐ I don't know ☐Yes; at what age ______

Medical Research Council Dyspnea Scale

I only get breathless with strenuous exercise ☐ Yes ☐ No

I get short of breath when hurrying on the level or walking up a slight hill ☐ Yes ☐ No

I walk slower than people of the same age on the level because of breathlessness, or have to stop for breath when walking at my own pace on the level ☐ Yes ☐ No

I stop for breath after walking 100 meters or after a few minutes on the level ☐ Yes ☐ No

I am too breathless to leave the house ☐ Yes ☐ No

Are you a current smoker (cigarette or narguileh)? ☐ Yes ☐ No

Part III: For Current Smokers

Do you currently smoke cigarettes? ☐ No ☐ Yes; If yes, please provide details

Number of cigarettes per day	Duration in years

Do you currently smoke narguileh? ☐ No ☐ Yes; If yes, please provide details

Number of narguileh per week	Duration in years

Part III: For Previous Smokers

Are you a former cigarettes smoker? ☐ No ☐ Yes; If yes, please provide details

Number of cigarettes per day	Duration in years

If yes, how many years ago did you stop smoking cigarettes? ___________ years

Why did you stop cigarette smoking? ____

Did you smoke narguileh previously? ☐ No ☐ Yes; If yes, please provide details

Number of narguileh per week	Duration in years

If yes, how many years ago did you stop smoking narguileh? ___________ years

Why did you stop smoking narguileh? ____

Please answer the following questions if you have asthma:

Do you visit the doctor for regular check-up? ☐ Yes ☐ No

How many times were you admitted to the emergency room this year for asthma? ______times

How many times did you visit the doctor this year for asthma? ______times

How many times were you hospitalized for more than one day for asthma? _____times

For how long have you been treated for asthma? _________

How do you evaluate your asthma condition?

☐ Totally controlled ☐ Partially controlled ☐ Not controlled ☐ I don't know

How many times did you use Ventoline/day in the past week?

☐ Once per day ☐ Twice per day ☐ 3 times per day ☐ 4 times per day

How many times did asthma prevent you from doing your daily activity in the past week?

☐ Every day ☐ 2 days or more ☐ One day ☐ Never

How many times did asthma prevent you from doing your favorite sport in the past week?

☐ Every day ☐ 2 days or more ☐ One day ☐ Never
